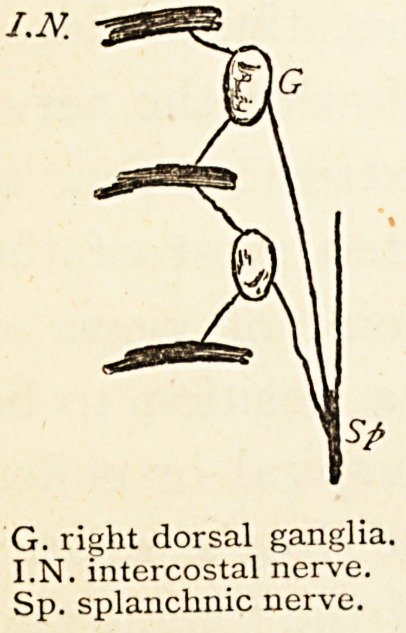# The Effects of Certain Anatomical Relations

**Published:** 1884-03

**Authors:** James Cantlie

**Affiliations:** Demonstrator of Anatomy and Senior Assistant-Surgeon, Charing Cross Hospital


### THE EFFECTS OF CERTAIN ANATOMICAL RELATIONS.

A Lecture delivered before the Medical Society, Charing Cross Hospital.

BY

JAMES CANTLIE, M.A., M.B., F.R.C.S.,

*Demonstrator of Anatomy and Senior Assistant-Surgeon, Charing Cross Hospital.*

---

MR. PRESIDENT AND GENTLEMEN,

It is my intention to give you a short epitome of some reflections, which, during a twelve years' course of anatomical teaching in this school, have occurred to me at different times. Most anatomical teachers have within their own store book of knowledge, unpublished thoughts and theories, which never see the light, but I have determined to set mine forth, criticise them as you may.

### ON THE RELATIVE WEIGHT OF THE RIGHT AND LEFT SIDES OF THE BODY.

It is believed and largely taught that the right side of the body is heavier than the left, and very pretty theories have been advanced accordingly as to the use of the right hand. The organ to be looked upon as yielding the predominant weight is the liver, which, weighing as it does 50 ozs., has only the spleen, weighing 7 ozs., to balance it; for the kidneys all but weigh the same; the pancreas lies a little more on the right than the left; the right lung is 2 ozs. heavier than the left; and the only other organ throwing weight into the left scale is the heart, which presents a little more on the left than the right side.

A table of proportionate weights yields this result:—

| | Right side. | Left side. |
| --- | --- | --- |
| Liver | $44\frac{1}{2}$ | $4\frac{1}{2}$ |
| Pancreas | $1\frac{3}{4}$ | $1\frac{1}{4}$ |
| Spleen | 0 | 7 |
| Lungs | 20 | 18 |
| Heart | $2\frac{1}{2}$ | $6\frac{1}{2}$ |
| Kidneys | $4\frac{1}{2}$ | $4\frac{1}{4}$ |
| | $73\frac{1}{4}$ ozs. | $41\frac{1}{4}$ ozs. |

Hence the right side according to this table is heavier than the left by $31\frac{3}{4}$ ozs.

Now what are the facts? On carefully dividing the thoracic and abdominal viscera, by a central incision from the top of the sternum to the symphysis pubis, it is found that the *left*, not the right, but the viscera on the *left* side of the body weigh $13\frac{1}{4}$ ozs. more than the right, *i.e.*, not only is the deficiency of $31\frac{3}{4}$ ozs. made up but there are $13\frac{1}{4}$ ozs. to the good. What is there then to make up this $31\frac{3}{4}$ ozs. + $13\frac{1}{4}$ ozs. = 45 ozs. upon the left side? There is the intestine, and it is the intestine, and chiefly the small intestine, which makes up this lee way. The small intestines lie chiefly on the left iliac fossa and also in the pelvis between the bladder and rectum. The effect of the first position is to compensate for the weight of the liver on the right and balance the body; and the effect of the latter position is to carry the focal point of the body weight below the centre of the axis of the weight of the body.

Here then is ground work for theorists to work on.

They will find it much more easy to explain how the light (right) side rotates forwards whilst the heavier (left) drags behind. They will also find food for discussion, in the fact that, the heavier side has its over-load low down below the centre of balance of our bodies, whilst the right has its over-load above that point, and which acting without compensation, would unbalance the body and lead to an unstable gait. How that there is a spiral idea in having the weights at different heights on opposite sides. They may even indulge so far as to account for the left foot being the one chosen to mark the time on a march to music. Whatever explanations may come out of it, these are the facts, that the left side is heavier than the right, and that the small intestines are the cause of this surplus of weight on the left side.

A general survey of the body viscera reveals the fact that the viscera containing air, and consequently the lightest, are seated the highest and above the diaphragm, whereas the heavier contents, fœces and urine, find their way from above downwards until they are met with in quantity at the lower part of the abdomen. The position of the stomach, liver and spleen so close beneath the diaphragm and up under cover of the cartilages of the ribs gives food also for much comment. In the first place it is to be noticed, that the stomach is but a weak-walled organ considering the amount of muscular work it has to do; that the bile flows through the liver in channels destitute for the most part of muscular fibres; and that the spleen, an organ of recesses and fibrous network, has few muscular fibres in its tissue. Birds have no diaphragm but have gizzards, and one cannot help associating the two facts and relegating to the diaphragm the function of helping a weak-walled stomach in its action. We are apt

Diagram of vertical section through the median line of body showing the relations of the diaphragm:—
Dia. diaphragm. L. liver. S. stomach. P. pancreas. C. colon. D. duodenum third part. I. small intestine. Ao. aorta. L.R.V. left renal vein behind duodenum.

to consider the diaphragm a transverse partition, but the accompanying diagram shows how much more perpendicular it is, and how it slopes down behind the liver and stomach. The spleen is also within its grasp, and so it comes about that the diaphragm not only descends, but also advances forwards, thus throwing the liver, stomach and spleen against the anterior wall of the abdomen. The tension of the muscles therein contained is overcome, and the wall advances; but during the process the three viscera mentioned are pushed against the wall and are thus compressed, the weak-walled stomach is thus aided in its action; the bile is helped along the non-muscular biliary capillaries and the blood is aided in its struggle through the open mesh-work of the spleen. Supposing the abdominal wall is held rigid whilst the diaphragm contracts, vomiting takes place, and soon bile finds its way into the stomach, showing that the stomach is compressed between the diaphragm and the abdominal wall and that the bile is squeezed out of the liver. These statements are known to all, and we find conviction follows without further argument. Besides, is it consistent with the nature of things that much of the force generated by the contraction of the diaphragm should be lost? If it can be made mechanically useful is it not a conservation of force to employ it? The descent of the diaphragm by its aspiration fills the thorax with both air and blood, and are the organs below to be denied any good therefrom? As it aids respiration and circulation so it aids digestion, and the three all-important functions of our vegetative life are made to harmonise and depend on each other through this common agent the diaphragm. I would go further and say that just as every cranny and nook of the lung is affected by the action of the diaphragm and is covered by pleura to allow of the motion consequent on that action, so is every organ in the abdomen which is covered by peritoneum affected by the same muscle.

There are numerous other points to be considered in the relations of the abdominal viscera, but space will not admit of entering into them in detail. In short, notice how the main mass of the small intestines, the heaviest piece of viscera in our bodies, rests between the bladder and rectum below the level of the central balance of our bodies. Notice also the position and structure of the colon. The ascending colon, so wide in its calibre and with walls incapable of perfect contraction, must have difficulty in its effort to drive its contents along. Its position would induce one to believe that it would be better filled during the horizontal position; and who, after being amused at the idea, will not be convinced that it is during sleep that the contents find their way up the ascending colon, and for that matter along the transverse colon during rest on the left side. On getting out of bed and assuming the erect position the fœces by gravitation are helped down the descending colon, and by the time breakfast is over the contents have arrived at the anus in the form of the " morning stool."

Again, notice the position of the third part of the duodenum and its relation to the left renal vein (see diagram). The vein is placed directly between the aorta and the duodenum; when the duodenum is full the blood in the vein must have rather a struggle to get along, and not only is the left so placed but the right renal vein is covered by the second portion of the duodenum. Can it be that these have to do with the renal congestion which ensues after each meal? Does the full duodenum cause an increased pressure on the renal veins and thence on the malpighian tufts, thus providing for elimination of fluid, &c.? Laugh at this first and think of it afterwards, and make a better explanation than my poor attempt, and although I have not convinced you, if I have stimulated you to think about such matters I have obtained all I wish.

At examinations it is a common question, "Do you see the first portion of the duodenum when you open the cavity of the abdomen?" The candidate, taught by his teacher to say yes, may be shown by the examiner that it is not so, but also *vice versa* may take place, so that it is not a settled question. Now the real fact is, that when the stomach is empty the duodenum in its first portion is to be seen, but when the stomach is distended the duodenum disappears under the liver. Not only so, but look exactly where the duodenum does disappear to, and you will be led along the gall bladder from fundus to neck, and the direction of the first portion of the duodenum when the stomach is full is seen to be parallel to the long axis of the gall bladder. Does this suggest anything? Would not the pressure exercised by these two on each other be greater when they are full than when empty? When are they full? The duodenum is full during digestion and the passage of food from the stomach. The gall bladder is

Relation of D. duodenum and S. stomach to G.B. gall bladder. A. when the stomach is empty; B. when the stomach is full.

being constantly filled between meals, but is emptied of its contents during digestion, and bile is found ready in quantity in the second portion of the duodenum to meet the food as it leaves the stomach. Hence during digestion they are both full; and the first portion of the duodenum, tucked under the liver by the forward swing of the greater curvature of the stomach when full, presses against the gall bladder from fundus to neck. Muscular fibres in the gall bladder are scarce, and it has always been a perplexing question how the gall bladder empties itself—galvanic stimuli exciting but slow and meagre contractions therein. Here is a means by which a full stomach and duodenum provide for the flow of their own bile, and the fuller these are the more will the gall bladder be pressed upon and consequently the more completely emptied. Why fly off to central nervous and reflex nervous influence, when a mechanical explanation is at hand? Is the mechanical less wonderful than any other explanation? It is usual to ascribe it to vital, nervous, reflex or any of the other cloaks of ignorant subterfuge which men take to when every other escape fails.

Has it ever struck you as anything peculiar that veins and arteries should run so persistently and so closely together? Has it ever occurred to you that one might derive help from the other? If so, it must be the strong which gives help to the weak, namely, the artery to the vein. The veins and arteries in the limbs more especially are enclosed in a common sheath; now the blood rushing along an artery would tend to fill the sheath and drive all the blood in the veins back the way it came. To prevent this, valves are inserted in the walls; and these, supporting the blood until such time as the arterial wave has gone on, keep the blood ready to occupy the spot where the pressure becomes negative. Thus the venous blood is piled up as it were by the current in the artery, and kept ready until the removal of the pressure allows its onward course. As an example of the effect of the relation of arteries to veins, take the common iliac arteries and veins. The veins and arteries on the front of the fifth lumbar vertebra are so placed that the veins are on a plain posterior to the arteries, and they seem plastered against the vertebral column. Now when the artery contracts there is less resistance to the passage of venous blood, but when the arterial wave is passing, the blood which is in the veins must be stemmed, and an accumulation will occur in the iliac veins of the left side, which accumulation, the moment the artery contracts, will allow for a larger rush of blood than would otherwise happen. Is it not possible, also, that the sudden expansion of the artery upon the full vein may give the blood in the vein a help and a flip, by which it will pass up the long valveless cava? That there is a difficulty for the venous blood to pass there, is to be made out by—

1. Trying to pass a dissecting-room blow-pipe along it when the arteries are distended with injection.

2. During disease, when phlegmasia dolens exists, many museum specimens show that a thrombus extends all the way up to, but not beyond, the crossing of the left common iliac vein, behind the right common iliac artery, thus helping the notion that if fluid blood found it difficult to pass, clotted blood found it impossible.

3. The consideration that phlegmasia dolens of the right lower extremity is more often fatal than upon the left, and as the venous blood in the right iliac veins can pass more easily than in the left, so also may a clot more easily pass and thus account for the phenomenon.

Space will not allow of a further prosecution of the consideration of the relation borne by arteries to veins, but if I have set you agoing thinking in that direction it is something.

I now wish to draw your attention to a subject which I discussed before you some six years ago. Whilst preparing a frog's sciatic nerve for a physiological demonstration, I noticed a peculiar, but very decided, spiral or coiled appearance of the components of the nerve trunk. On stretching the nerve the coiling disappeared, but on relaxation it appeared again. I, at that time said, I believed it had something to do with allowing the nerve to accommodate itself to the flexion, extension, &c., of the hip, and since then I have inspected most of the nerves in the human body from this point of view. I find now, that all nerves which are in a position to be stretched, present the same (naked eye) spiral or coiled appearance, as I met with first in the frog's sciatic. Beautifully marked is it in the nerves of the tongue and penis; in these structures one would expect to see it if it existed, owing to the frequent variations they undergo in length. In the nerves of the bull's penis I found it beautifully marked now some four years ago. Nerves running through long canals, such as the superior maxillary nerve in its course through the infraorbital canal, do not show it. So, the nerves of the hand and foot, as they approach the end of their course lose the appearance; the median and ulnar about half way down the fore-arm cease to show it, and the nerves of the leg, markedly the anterior tibial, are destitute of any such condition. The nerves mentioned are obviously not so likely to be affected by the limited motions of the wrist and ankle as are those crossing the knee and elbow. Hence almost all the trunks of the nerves in the extremities as low as the knee and elbow, show it well. To begin with,—let me advise you to look for the condition mentioned in the sciatic nerve and the cords of the brachial plexus; and you will at once see what I mean. I intend soon to give you a table of the nerves in which this condition is to be met with.

Before finishing, let me draw your attention to another fact that has often attracted my attention. It is this:—On fixing your attention on one of the dorsal ganglia of the sympathetic, and then moving the head of the rib, it will be seen that the ganglion is affected by the motion of the rib; it is pressed upon alternately at its upper and lower part as the rib rises and falls. Also observe that when two nerves leave a ganglion to join the intercostal nerves, one goes from the upper part of the ganglion to the nerve above, the other from the lower part of the ganglion to join the intercostal nerve below. Hence the upper and lower part of each dorsal ganglion are affected alternately by the upward and downward movement of the ribs, and each intercostal nerve receives a filament during both actions. Observe also it is only in the thorax, where the ganglia lie so far back, in the abdominal region especially they come forward to near the middle line. Why do these ganglia lie so far back in the dorsal region? Is it because of giving filaments to the intercostal nerves? No! the lumbar ganglia give similar branches, and they are connected by nerves three or four inches long. Besides these branches to join intercostal nerves, splanchnic nerves supplying the liver and all the upper abdominal viscera arise from the

G. right dorsal ganglia.
I.N. intercostal nerve.
Sp. splanchnic nerve.

dorsal ganglia. Are there not some physiological and clinical observations about the glycogenic function of the liver being interfered with, when the ribs, with which these ganglia and nerves are associated, are broken? In other words is this communicated movement purposeless, is this tilting of the ganglia useless, is the course of the dorsal ganglia over the heads of the ribs accidental? That dare not be said; and one is driven to the conclusion that some purpose is served by it, be that a mechanical stimulus by which inhalation prompts exhalation, or *vice versa,* as the upper and lower part of the ganglia and so the intercostal nerves are alternately affected; or is it that some useful stimulus is conveyed to the splanchnic nerves as they proceed to the abdominal viscera?

I am much obliged to you for the way you have listened to these few points which have occurred to me, and whilst hoping they may be interesting to you to consider, I hope at some future time to continue the subject in greater detail.

## Figures and Tables

**Figure f1:**
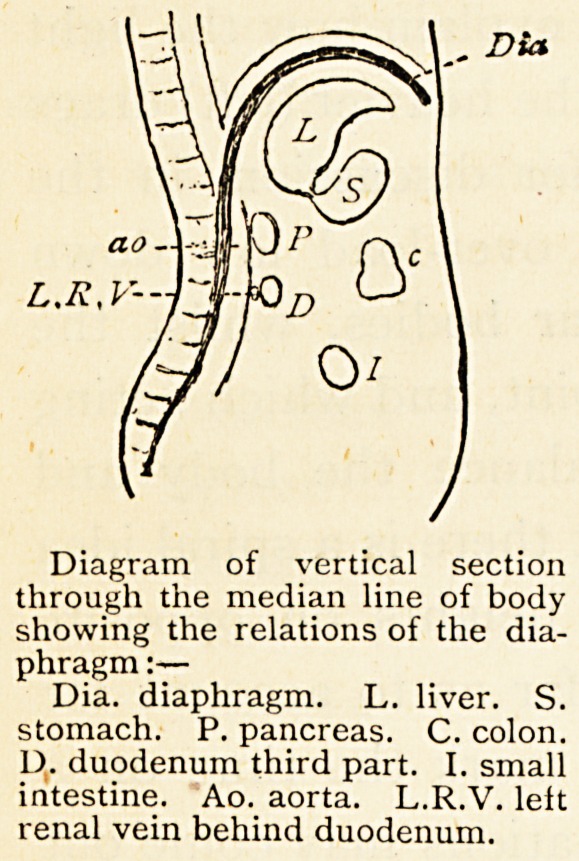


**Figure f2:**
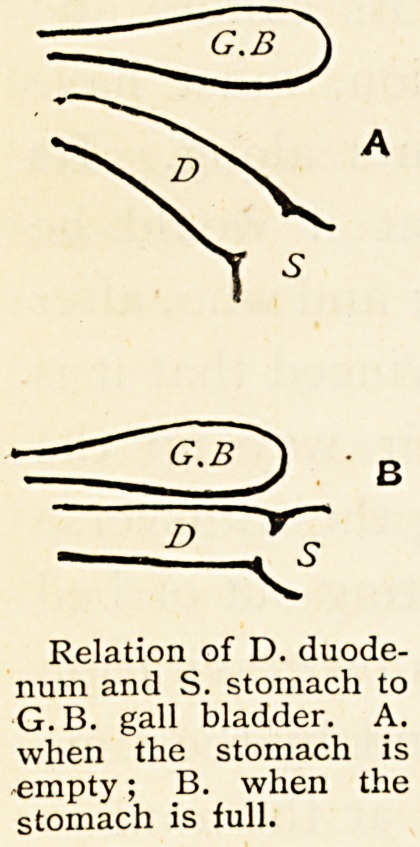


**Figure f3:**